# Hydrostatic pressure mapping of barium titanate phase transitions with quenched FeRh

**DOI:** 10.1038/s41598-020-63358-0

**Published:** 2020-04-14

**Authors:** Christian Urban, Steven P. Bennett, Ivan K. Schuller

**Affiliations:** 1 Department of Physics and Center for Advanced Nanoscience, University of California, San Diego, La Jolla, California, 92093 USA; 2 U.S. Naval Research Laboratory, Washington, DC USA

**Keywords:** Materials science, Physics

## Abstract

We report a pressure study of the metamagnetic/ferroelectric hybrid heterostructure of a quenched FeRh thin film (25 nm) grown on single crystal barium titanate (BTO). It has been previously reported that when the BTO undergoes a crystal transition a massive magnetization and coercivity change is triggered in the highly strain sensitive quenched FeRh thin film. Therefore quenched FeRh makes for an ideal probe for mapping a materials structural phase transitions. In this work we demonstrate this effect as a function of both temperature and hydrostatic pressure. As a result, we present the pressure dependence of the hybrid material which aligns identically with the BTO substrates pressure dependence reported in literature. The concept of combining a structural phase transitional (SPT) material with a magnetostrictive magnetic metal has been shown with vanadium oxides and our findings here prove that this methodology can be extended to strain sensitive metamagnetic materials systems in thin film, and possibly in bulk, heterostructures.

## Introduction

Interfacial proximity effects provide one strategy to explore new hybrid materials in which one material component is used deliberately to control another^1^. New materials can therefore be designed intelligently for a specific functionality, or to answer basic fundamental questions. For the latter, one material with a relevant property is brought into intimate contact with another material which exhibits a control handle. In this case, and also in other examples^2,3^, the first order structural phase transition (SPT) enables external control through temperature. Additionally, since the SPT temperature is also sensitive to strain, pressure itself can be used as external control over the whole hybrid material.

In previous publications this approach has been shown successfully with the example systems of magnetic materials on SPT vanadium oxides, and a proof-of-concept has been provided that this can be extended from thin films to bulk^4^. The first report of its use was reported by T. Saerbeck *et al*. in 2014 to show an appreciable change in the magnetization of a Ni film on bilayers of VO_2_ and V_2_O_3_^1^. In this work, a Ni film is grown on top of VO_2_/V_2_O_3_. The phase transitions for both of these phases coincide with volumetric strain as well as the typically known metal to insulator transition. This volumetric strain drives the magnetostriction effect in the Ni film which can easily be detected by magnetometry. Using magnetometry as a detection method opens the door to laboratory based measurements of the pressure induced properties of the SPT, where-as in the past such a measurement would require the use of neutrons to measure d-spacing changes in a pressure cell. Other similar works have followed T. Saerbeck for vanadium oxide hybrids in thin film and bulk^1,4–8^, however in this work we take this scheme a step further by not relying solely on the weak magnetostriction effect in ferromagnetic films to detect the transition, and instead reveal that the strain sensitive transition in metamagnetic FeRh, combined with its magnetoelasticsensitivity, is a strong tool for studying these responses. These methods are vital for our continued understanding of SPT in these systems as they are of high technological relevance for heat assisted magnetic random access memories (HA-MRAM)^9,10^.

In this work we focus on a different and unique SPT-material; barium titanate (BTO). The three first order SPTs are sensitive to pressure similar to vanadium oxide^11^, and together with the intrinsic ferroelectricity, BTO offers several ways to control a material in close proximity^5,12^. Combining BTO with a magnetic compound with a reasonable magnetostrictive coefficient offers a way to control this hybrids magnetism without external fields. The SPT temperature is sensitive to pressure which therefore becomes an additional means of control over the second materials magnetism. A two-state magnetization and coercivity system with a well defined, variable temperature in a wide window can be fabricated if the chosen magnetic component is at least partially ferromagnetic throughout the relevant temperature range.

While a highly epitaxial homogeneous single phase of FeRh would undergo a transition from anti-ferromagnetic to ferromagnetic at ~360 K, our quenched FeRh (q-FeRh) shows a highly ferromagnetic response throughout the temperature regime studied in this publication, (detailed further in the Supplementary Material). Furthermore, FeRh has been in the focus of material research for its magnetic transition^13–18^ and other device relevant properties^19–22^. Combining both materials, BTO and FeRh in a thin film hybrid, creates a material with several ways to control the magnetic characteristics. Extrapolating the findings from the literature about Ni/V_2_O_3_ pellets^4,6^, it is likely that the obtained control shown in this publication also be transfered from the thin film to the bulk FeRh/BTO. Note that an additional way to control the hybrid characteristic is using the ferroelectricity which potentially can alter material properties similar to other material systems^23^.

**Figure 1:**
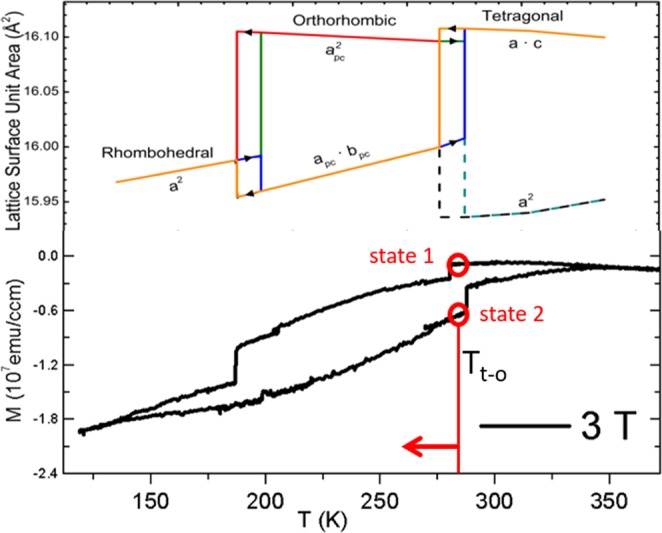
The structural changes at the SPTs triggers the saturation magnetization change (B = 3 T) in q-FeRh/BTO thin films. Only two (190 K and 280 K) of three SPT regions (390 K) are displayed here in one graph due to the large temperature window at which they occur. Two distinct states (red circles) can be found at the center of the hysteresis close to one of the transition temperatures (T_t-o_) at which increasing external hydrostatic pressure shifts the transition temperature to lower temperatures (arrow). M vs. T data was taken with a 3 T applied field.

We have shown previously that q-FeRh undergoes large magnetization changes (Fig. 1) driven by the BTO’s SPT^23^ including a two-state magnetization at the same temperature (Fig. 1 circles). In the present publication we provide evidence that the BTO also controls the coercivity in three different temperature regions associated with the temperature T_c_ of the corresponding SPT. Therefore this hybrid material exhibits three, two-state magnetizations and also three, two-state coercivities at the different T_c_’s of BTO. Our results prove that the magnetic hybrid properties can be tailored with pressure. T_c_can then be inferred from the p-dependence of the substrate material. This also shows that the q-FeRh/BTO hybrid resembles exactly the BTO P-dependence. We assume that this is valid for all three SPT’s although we only show data of the pressure dependence of two of the three SPT’s of the substrate. These are the tetragonal-to-orthorhombic and the cubic-to-tetragonal transition located at T_t-o_ = 290 K and T_c-t_ = 390 K, respectively^24^. With less than 4 GPa total pressure (with possible components from chemical, external and built-in pressures) the temperature at which a two-state coercivity or magnetization will be located can be tailored semi continuously in the wide range of 150–390 K (see Supplemental Material for details). The knowledge of the substrate pressure dependence provides therefore a predictable way of tailoring new magnetic hybrid materials based on the proximity effect and a SPT compound.

This does not only render our hybrid material a perfect candidate for a two-state magnetic device controlled by pressure or by potentially exploiting the ferroelectric properties of BTO^5,12,25–27^, but at the same time shows the working concept of using FeRh as the magnetic probe material to determine structural change dependencies of a SPT materials with FeRh on top. These hybrids can then be exploited in tailored devices of new material combinations.

## Experimental

In order to achieve a partial ferromagnetic response of FeRh in the relevant temperature regime, the growth protocol for the 25 nm FeRh thin films included a rapid quenching after a sputter deposition from a 50:50 composition target on the BTO substrate. The unique properties and fabrication details of such samples have been reported in a previous publication^23^. We confirmed that the samples show ferromagnetic hysteresis loops between 150 K and 400 K (see SM) and are therefore suitable as a probe for the BTO phase transitionsin in that range. These take place at T_o-r_ = 190 K, T_t-o_ = 290 K andT_c-t_ = 390 K^24^.

**Figure 2:**
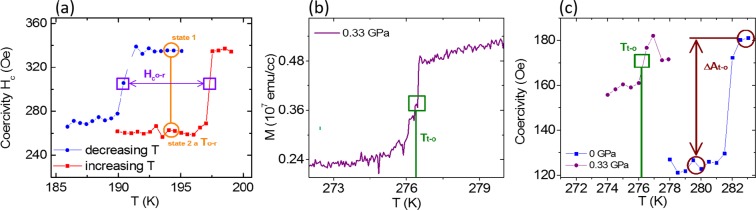
(**a**) Coercivity (Hc) as a function of Temperature (T) at ambient pressure of the orthorhombic to rhombohedral (o-r) SPT. The empty circles indicate the two-state coercivity at the same temperature while the empty squares indicate the transition temperature for the different temperature sweep directions (also from where the average transition temperature T_o-r_ and the hysteresis width H_o-r_are derived). b) For clarity only the increasing temperature path is shown for two pressures of the tetragonal to orthorhombic transition.The coercivity change is indicated with the open circles. The position of the open squares indicates the transition temperature for the increasing T path at the corresponding pressure. That can be compared to c) where the magnetization response at the same SPT in the sensitive remanence state for the same pressure as in b) is shown.T_t-o_is defined as the half way point of the curve through the magnetization change (open square) of the increasing temperature path and is the same for both methods.

The structural change (~1% volume change) of the BTO substrate, while sweeping the temperature through any given phase transition, creates strain in the deposited thin film in close proximity. This manifests in changes of the magnetization and the coercivity in the ferromagnetic portions of the heterostructure due to the inverse magnetostrictive effect^28–33^, as well as potentially triggering the metamagnetic transition in the strain sensitive antiferromagnetic domains. We measured directly both of these two magnetic properties in the low pressure regime (0–0.33 GPa). The changes during the SPTs correlate with changes in a special remanence state (also denominated “counter field measurements”, see SM). This state is more sensitive to the SPT triggered by the temperature change while sweeping through the relevant temperature window and is therefore a good method to track the changes associated with the SPTs of BTO at higher pressures where the signal-to-noise ratio is smaller for measurements of H_c_. The protocol for this method consists in: 1. saturating the magnetization of the sample in the positive direction with 0.6 T, 2. applying a small counter field in the negative field direction of – 0.01 T and 3. sweeping the temperature for the decreasing (increasing) temperature direction from above (below) the transition temperature to below (above) the transition while measuring the magnetization. The latter (Fig. 2a) and also the saturation magnetization (Fig. 1) show a clear increase or decrease across the SPTs of BTO. The temperature difference ΔT between the T_c_’s of the two sweep directions (increasing and decreasing T) is equivalent to the hysteresis width of the SPT measured at the center of the transition. We have mapped with this method  the T_c_ of the tetragonal-to-orthorhombic STP at 280 K, denominated “T_t-o_”, and the cubic to tetragonal SPTat 390 K, denominated “T_c-t_”, for the pressure range of 0 to 1.5 GPa. In order to change the pressure we have used a commercial pressure cell placed inside a standard magnetometer^34^ which allows us to apply hydrostatic pressures on our samples of up to 1.5 GPawhile measuring the magnetization.

## Results

From standard magnetic hysteresis loops at different T and zero pressure we obtained the coercivity as a function of temperature (Fig. 2a) and the transition temperature at each SPT as the center temperature between the increasing and decreasing temperature paths:

T_t-o_  = (T_t-o_,_increasing_ − T_t-o_,_decreasing_)/2 + T_t-o_,_decreasing_ as indicated in Fig. 2a as the position of open squares. This hysteretic behavior is the reason why there are two-states for the magnetization and the coercivity at approximately the transition temperature (see also the empty circles in Fig. 1 and Fig. 2a, respectively).The two-state magnetization and coercivity can possibly be exploited in memory devices and transformer cores, respectively.

 The maximum coercivity changes at zero pressure, occurring while sweeping the temperature through the three SPTs (from high to low T),  are ΔA_c-t_ = 40%, ΔA_t-o_ = −53% and ΔA_o-r_ = −46% (see the open circles in Fig. 2b).

**Figure 3:**
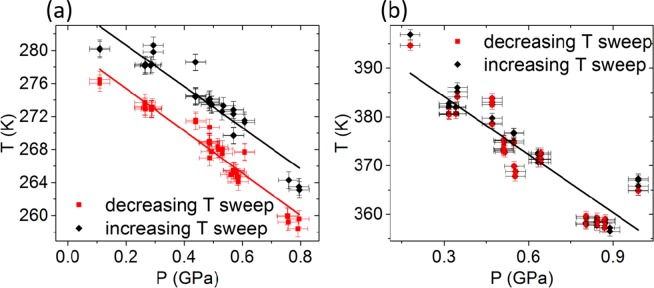
The transition temperatures at BTO’s *tetragonal to orthorhombic* phase transition (T_t-o_) in (**a**), and BTO’s *cubic to tetragonal*(T_c-t_) in (**b**). Both plotted as a function of hydrostatic pressure for increasing (red squares), and decreasing (black diamonds), temperature sweep directions.

The transition temperatures as a function of pressure are obtained by increasing step wise the pressure and measuring the remanence state as can be seen in Fig. 3 which shows the experimental data points for the SPT close to RT, T_t-o_, in Fig. 3a, and for the SPT close to 390 K, T_c-t,_ in Fig. 3b. The transition temperatures change linearly with P and the dependence of both SPTs on pressure is different and consistent with the literature values of BTO bulk^35–37^. We found at 280 K: **dT**_**t-o**_**/dp** = **−25** ± **2 K/GPa** and at 390 K: **dT**_**c-t**_**/dp** = **−40** ± **2 K/GPa**.

The hysteresis width is defined by the difference of the center of the magnetization change for both temperature sweep directions. It is also different for all the cases and:

$${{\rm{DT}}}_{{\rm{o}}-{\rm{r}}}=7.5\pm 0.8{\rm{K}},{{\rm{DT}}}_{{\rm{t}}-{\rm{o}}}=4.4\pm 0.7{\rm{K}}\,{\rm{and}}\,{{\rm{DT}}}_{c-{\rm{t}}}=1.5\pm 0.4{\rm{K}}.$$

As a comparison, the pressure dependence of the FeRh transition in bulk is positive. It shifts with higher pressures to higher temperatures contrary to the BTO transitions and is roughly twice as sensitive to pressures with dT_c,FeRh_/dp~+45 K/GPa^38^. Furthermore, the FeRh transition is usually very broad and takes place over several tens of K different to the observed transitions which are very sharp with a width smaller than 5 K. Therefore any confusion of this transition with our measurements can be excluded.

The low temperature transition data of BTO around 190 K shows also a two-state magnetization and coercivity (Figs. 1 and 2a, respectively) but has not been measured as a function of pressure. We assume the pressure dependence is also consistent with literature values for BTO bulk material given that the dependence of the two other transitions support that assumption and the two-state properties would therefore shift with dT_o-r_/dp = −12 K/GPa^39^.

## Conclusions

We have created a tunable two-state magnetization and coercivity material based on q-FeRh/BTO heterostructure. This unique material combination is an example of a novel, tunable, hybrid compounds based on a magnetostrictive film grown on a SPT material. Using pressure, the temperature locations of the two-state magnetizations and coercivities have been shown here to be tunable semi-continuously in a very large temperature regime.

The measured pressure dependencies for the presented hybrid are consistent with BTO literature values and render our hybrid-concept predictable in terms of the temperature location for the two-state magnetization and coercivity. Pressure sources to control the hybrid are hydrostatic pressure but can also be intrinsic strain by mismatch growth or, in the case of BTO, possibly using piezoelectricity.

Additionally, we have found that q-FeRh is a good thin film probe to test SPT materials. The requirements for that are: 1. intimate contact with the first order SPT material and 2. a volume change of the SPT material of approximately 1% during the transition(s). The first requirement ensures that the interface strain due to the volume change of the SPT material translates into the thin magnetic layer. Whereas the second is necessary to create a structural strain big enough so that the magnetostrictive material, here q-FeRh, will show a property change. FeRh exhibits a sufficiently high magnetostrictive coefficient and the SPT material will conditions trigger a change in the magnetization and coercivity due to the SPT(s).

It is noteworthy that this concept is not limited to thin film systems but also applies to mixing intimately two bulk materials, one exhibiting ferromagnetism and the other with at least one SPT^4,6^. Moreover SPT’s can be triggered with light^40,41^, current injection^42^, electric fields^43–46^ and more^12,27^, which provide an additional control mechanisms over hybrids of potential interest for data storage, transformer cores and antiferromagnetic/metamagnetic electronic devices.

## Supplementary information

Supplementary information.
