# Supplementary material for: Hydrostatic pressure mapping of barium titanate phase transitions with quenched FeRh
Source: Sci Rep. 2020 Apr 14;10:6312. doi: 10.1038/s41598-020-63358-0 (PMC7156734; doi:10.1038/s41598-020-63358-0)
Supplement: Supplementary file 1 — Supplementary information. [file 41598_2020_63358_MOESM1_ESM.docx]

Supplementary material for “Hydrostatic pressure mapping of barium titanate phase transitions with quenched FeRh”

Christian Urban^1^, Steven P. Bennett^2^ and Ivan K. Schuller^1^

^1^ Department of Physics and Center for Advanced Nanoscience, University of California, San Diego, La Jolla, California 92093, USA

^2^U.S. Naval Research Laboratory, Washington DC

**S1: Pressure determination:**

The pressure inside the cell was measured using the transition temperature of a small piece of lead. At zero pressure it was 7.170K. Then with the pressure dependence of lead from literature [1-2]: dT_c,Pb_/dp = -0.365 ± 0.003K/GPa, the pressure at T_c,lead,p_ could be determined. In order to extract the pressure at the temperatures of the substrate’s STP’s, experimental data of the pressure dependence of the pressure medium used (Daphne 7373) was used to extrapolate [3]. The pressure uncertainty is smaller than 5%.

**Evidence for partial ferromagnetism throughout the relevant temperature regime:**

f)

e)

d)

c)

b)

a)

a)

Figure S1: Ferromagnetic loops at zero pressure at different relevant temperatures below and above the three different transitions. Figure S1(a) shows an M v H hysteresis at T = 185K which is below the BTO rhombohedral to orthorhombic SPT transition. Figure S1(b) shows an M v H hysteresis taken at T = 197K which is above the rhombohedral to orthorhombic BTO SPT. Figure S1(c) shows M v H taken at T = 270K which is just below the BTO orthorhombic to tetragonal SPT. Figure S1(d) shows an M v H taken at T = 289K which is above the BTO orthorhombic to tetragonal SPT. Figure S1(e) shows an M v H taken at T = 395K which is just below the BTO tetragonal to cubic SPT. Figure S1(f) shows an M v H taken at T = 400K which is above the BTO tetragonal to cubic SPT. Together these show that FeRh quenched on BTO is partially ferromagnetic throughout the T regime close to the SPT’s.

The temperature regime at which the SPT’s of BTO occur is partially ferromagnetic as can be seen in Fig. S1. It shows hysteresis loops which reveal the ferromagnetic character of the sample in the window from 120 to 400K. The two state coercivity and magnetization can therefore be tuned with pressure in that temperature range.

1. **Correlation between coercivity and counter field measurements changes**

b)

a)

d)

c)

Figure S2: A comparison between the counter field measurement to improve the sensitivity (solid lines) and the coercivity measurement (dots). For all three SPT’s the coercivity and the magnetization change (counter field measurements) occur at the same temperature which renders the counter field measurement a more reliable way (more data points and less noise) to determine the temperature at which the SPT occurs. a-c) ambient pressure and increasing temperature path. This still holds for increased pressured which can be seen in d) 0.33GPa. Therefore we chose to employ the counter field method to determine the temperature dependence of the SPT since the signal to noise ratio is better.

Figure S2 shows data of all three SPT’s of the FeRh/BTO hybrid. The measured coercivity change correlates very well with the change in magnetization of the sensitive remanences (counter field) state. In c) and d) data for the same SPT but two different pressures are shown and confirms the correlation.

1. **Continuous tunability of two-state temperature**

In order to shift the o-r SPT of BTO 40K down from 190 to 150K, less than 4GPa is necessary (3.4GPa), based on the measured pressure dependence for that transition (-12K/GPa). A pressure dependence of 25K/GPa requires 3.6GPa to shift the RT-SPT from 280 to 190 K and would shift the highest SPT from 390 all the way down to 246K. Therefore with 3 GPa a two-state coercivity or a two-state magnetization can be created and utilized anywhere between 150-390K in this hybrid sample. This is without any intrinsic strain/pressure (mismatch growth) or chemical pressures (doping) which could be applied to reduce the external pressure needed to shift the two-state coercivity and magnetization or to increase further the temperature range for a two state system.

REFERENCE:

1 M. J. Clark, T. F. Smith, Pressure dependence of Tc for lead, J. Low Temp. Phys. 32, 495-503 (1978).

2 A. Eiling, and J. S. Schilling, Pressure and temperature dependence of electrical resistivity of Pb and Sn from 1-300K and 0-10 GPa-use as continuous resistive pressure monitor accurate over wide temperature range; superconductivity under pressure in Pb, Sn and In, J. Phys. F: Met. Phys. **11**, 623-639 (1981).

3 K. Murata, H. Yoshino, H. O. Yadav, Y. Honda, and N. Shirakawa, Pt resistor thermometry and pressure calibration in a clamped pressure cell with the medium, Daphne 7373, Rev. Sci. Instrum. **68** 2490-2493 (1997).
